# The N17 domain mitigates nuclear toxicity in a novel zebrafish Huntington’s disease model

**DOI:** 10.1186/s13024-015-0063-2

**Published:** 2015-12-09

**Authors:** Matthew B. Veldman, Yesenia Rios-Galdamez, Xiao-Hong Lu, Xiaofeng Gu, Wei Qin, Song Li, X. William Yang, Shuo Lin

**Affiliations:** Department of Molecular, Cell and Developmental Biology, University of California-Los Angeles, 621 Charles E. Young Drive South, Los Angeles, CA 90095 USA; Center for Neurobehavioral Genetics, Semel Institute for Neuroscience and Human Behavior, Los Angeles, USA; Department of Psychiatry and Biobehavioral Sciences, David Geffen School of Medicine, Los Angeles, USA; Brain Research Institute, University of California, Los Angeles, Los Angeles, CA 90095 USA; Laboratory of Chemical Genomics, School of Chemical Biology and Biotechnology, Peking University Shenzhen Graduate School, Shenzhen, 518055 China

**Keywords:** Cre inducible, Huntingtin, Huntington’s disease, Polyglutamine, Zebrafish

## Abstract

**Background:**

Although the genetic cause for Huntington’s disease (HD) has been known for over 20 years, the mechanisms that cause the neurotoxicity and behavioral symptoms of this disease are not well understood. One hypothesis is that N-terminal fragments of the HTT protein are the causative agents in HD and that peptide sequences adjacent to the poly-glutamine (Q) repeats modify its toxicity. Here we test the function of the N-terminal 17 amino acids (N17) in the context of the exon 1 fragment of HTT in a novel, inducible zebrafish model of HD.

**Results:**

Deletion of N17 coupled with 97Q expansion (mHTT-ΔN17-exon1) resulted in a robust, rapidly progressing movement deficit, while fish with intact N17 and 97Q expansion (mHTT-exon1) have more delayed-onset movement deficits with slower progression. The level of mHTT-ΔN17-exon1 protein was significantly higher than mHTT-exon1, although the mRNA level of each transgene was marginally different, suggesting that N17 may regulate HTT protein stability in vivo. In addition, cell lineage specific induction of the mHTT-ΔN17-exon1 transgene in neurons was sufficient to recapitulate the consequences of ubiquitous transgene expression. Within neurons, accelerated nuclear accumulation of the toxic HTT fragment was observed in mHTT-ΔN17-exon1 fish, demonstrating that N17 also plays an important role in sub-cellular localization in vivo.

**Conclusions:**

We have developed a novel, inducible zebrafish model of HD. These animals exhibit a progressive movement deficit reminiscent of that seen in other animal models and human patients. Deletion of the N17 terminal amino acids of the huntingtin fragment results in an accelerated HD-like phenotype that may be due to enhanced protein stability and nuclear accumulation of HTT. These transgenic lines will provide a valuable new tool to study mechanisms of HD at the behavioral, cellular, and molecular levels. Future experiments will be focused on identifying genetic modifiers, mechanisms and therapeutics that alleviate polyQ aggregation in the nucleus of neurons.

**Electronic supplementary material:**

The online version of this article (doi:10.1186/s13024-015-0063-2) contains supplementary material, which is available to authorized users.

## Background

Huntington’s disease (HD) is an incurable, autosomal dominant neurodegenerative disease. Patients with HD display progressive symptoms including psychiatric, cognitive, and motor dysfunction, and the disease onset is often defined by the onset of motor symptoms [1–3]. Initially, patients present with excessive movements of the extremities and face, progressing to larger movements described as chorea. Further disease progression results in the opposite effect with patients suffering from rigidity, akinesia, bradykinesia, and gait abnormalities. The disease is invariably lethal 10–20 years after onset. There is no cure for HD and treatments for the symptoms are of limited effectiveness.

HD is part of a broader class of neurological disorders linked to polyglutamine (polyQ) repeat expansions within several disease proteins [4]. All of these disorders are progressive, degenerative diseases with age of onset inversely correlated with the number of polyQ repeats within the protein. Although the genes involved in polyQ diseases are, for the most part, broadly expressed, each disease has a well-defined pattern of neurodegeneration affecting selective populations of neurons. In HD, the medium spiny neurons (MSNs) of the striatum are thought to be most susceptible to degeneration and cortical pyramidal neurons are also affected [5]. The precise mechanisms underlying such selective neuronal vulnerability in HD remain incompletely understood. One hypothesis is that amino acid sequences flanking the polyQ repeat dictate the cell-type specific toxicity.

HD is caused by an expanded polyQ within the N-terminus of the huntingtin protein (HTT) [6]. HTT is a large, 3144 amino acids, protein that is ubiquitously expressed, evolutionarily conserved, and has been suggested to function as a scaffolding protein for many cellular processes. Knockout of this gene is early embryonic lethal in mice suggesting an essential developmental function [7–9], however the specific mechanisms involved are not well understood. Histologically, a hallmark of HD is large intracellular polyQ protein aggregates [10]. These aggregates can be localized to several sub-cellular regions including cytoplasmic, peri-nuclear, and intra-nuclear [11, 12]. Although originally thought to be disease causative, polyQ aggregates have been suggested to be neuroprotective [13] and their role in the disease is still unresolved. One hypothesis is that localization of the aggregate defines its toxicity. Experimental targeting of HTT-polyQ to different subcellular compartments suggests that the nucleus is especially sensitive to HTT-polyQ expression [14, 15]. The normal subcellular distribution of HTT is mediated by protein-protein interactions to regions outside the polyQ [16]. Most notably, the N-terminal 17 amino acids of HTT (N17) have been suggested to regulate protein localization and stability [17]. This region of the protein is evolutionarily conserved [18]. It functions as both a nuclear export signal and cytoplasmic membrane association domain [19, 20]. Additionally, its amphipathic alpha-helical structure [21, 22] promotes oligomer formation and accelerates HTT-polyQ fragment aggregation in vitro [23, 24]. Post-translational modifications of N17 are prevalent and diverse [25]. Phosphorylation of serines 13 and 16 has been demonstrated to reduce aggregation and toxicity in vitro [26] and in vivo [27]. In vivo, BAC transgenic mice expressing human mHTT-97Q lacking the N17 domain, a deletion of 2–16 amino acid residues in otherwise full-length mHTT context (BACHD-∆N17), results in greatly accelerated motor deficits including adult-onset movement disorder, striatal neurodegeneration, and exclusive nuclear mHTT aggregation formation [28]. The phenotypes of BACHD-∆N17 mice is dramatically accelerated and more closely resemble clinically-manifest HD than the BACHD mouse model expressing intact full-length mHTT of the same polyQ length [28, 29]. Therefore N17 acts as a modulator of mHTT aggregation, subcellular localization, and neurotoxicity.

Since the treatments available for HD are quite limited, it is important to have preclinical models of the disease that can be used to study mechanism and test candidate therapeutics. Mouse models are the most popular and have been developed over the last 18 years using conventional transgenic, YAC and BAC transgenic, and gene knock-in technologies to introduce polyQ into the genome [30]. Some models express the entire protein while others only use exon 1 which encodes the pathological polyQ expansion. These mice develop behavioral abnormalities and brain pathologies that recapitulate HD to varying degrees. Recent data suggests that proteolytic fragments containing exon 1 [31] or aberrantly spliced transcripts of exon 1 [32] are toxic and exist in patients. mHTT exon 1 based transgenic mice [33] have already been demonstrated to exhibit behavioral and pathological similarities to human disease, mHTT exon 1 based models are likely to be relevant to aspects of the disease. However, mammalian models are expensive to maintain and have limited scalability. Several non-mammalian models of polyQ toxicity have been developed in organisms such as *S.cerevisiae* [34], *c.elegans* [35, 36], and *Drosophila* [37]. These models are scalable for screening compounds and genetic interactions, but lack high genetic similarity to humans and have significantly different, or in the case of *S. cerevisiae* no, nervous system. Zebrafish are an advantageous vertebrate model organism that is genetically more closely related to humans than non-vertebrate models but is still scalable and reasonably affordable as compared to mammalian models [38]. HTT-polyQ toxicity has been reported in zebrafish by using mRNA or plasmid DNA injection to acutely over-express the protein [39, 40]. However, this model might not recapitulate specific mechanisms of the disease due to its early developmental effects and the extreme levels of protein expression that are necessary to cause toxicity. A second zebrafish model of polyQ toxicity has been reported in which the rhodopsin promoter drives mHTT-exon1 fragment expression in photoreceptors of the retina [41]. These zebrafish exhibit specific cellular degeneration and protein aggregation in the rod photoreceptor layer of the retina. However, retinal degeneration is not a known pathology in HD. Therefore, a zebrafish model that more closely recapitulates aspects of the human disease would be a valuable new tool for the field.

We have generated a series of conditional transgenic zebrafish models of HD. Using Cre*-loxP* technology, we have generated inducible transgenic fish that express HTT-exon1(25Q)-EGFP or mHTT-exon1(97Q)-EGFP upon Cre recombination. We have also generated complementary HTT-ΔN17-exon1(25Q)-EGFP and mHTT-ΔN17-exon1(97Q)-EGFP lines. These latter models were created to test if the accelerated nuclear pathogenesis and disease-like phenotypes observed originally in BACHD-∆N17 mice [28] could also be seen in our zebrafish model, and to test if N17 plays a crucial role in modifying the toxicities of mHTT-exon1, a disease-relevant toxic fragment in HD [32]. Upon ubiquitous recombination, EGFP^+^ protein aggregates are visible within both mHTT-exon1 and mHTT-ΔN17-exon1 lines. Surprisingly, these fish develop normally up to five weeks of age at which point mHTT-ΔN17-exon1 lines begin to exhibit abnormal movement and swimming behavior that progressively worsen until the fish are unable to swim by about 12 weeks of age. The mHTT-exon1 lines present much milder swimming impairment that does not appear until 4 months of age and progresses much more slowly. Additionally, we crossed the mHTT-ΔN17-exon1 line into transgenic Cre driver lines for neurons, glia, muscle, or vasculature. Only fish expressing mHTT-ΔN17-exon1 specifically in neurons developed a progressive movement disorder. Finally, we examined the subcellular localization of mHTT-exon1 fragments in the transgenic fish and found that mHTT-ΔN17-exon1 is enriched in the nucleus of neurons, providing direct evidence that N17 is crucial for cytoplasmic targeting/nuclear export of mHTT-exon1 in vivo and further suggests the nuclear toxicity is key to the manifestation of disease-like phenotypes in an HD vertebrate model. This new zebrafish model of HD will be an invaluable tool to further dissect the mechanisms of mutant polyQ toxicity and to screen for potential therapeutics or genetic modifiers to ameliorate disease-like phenotypes in vivo.

## Results

### Cre-*loxP* inducible expression of human HTT exon 1 in transgenic zebrafish

**Fig. 1:**
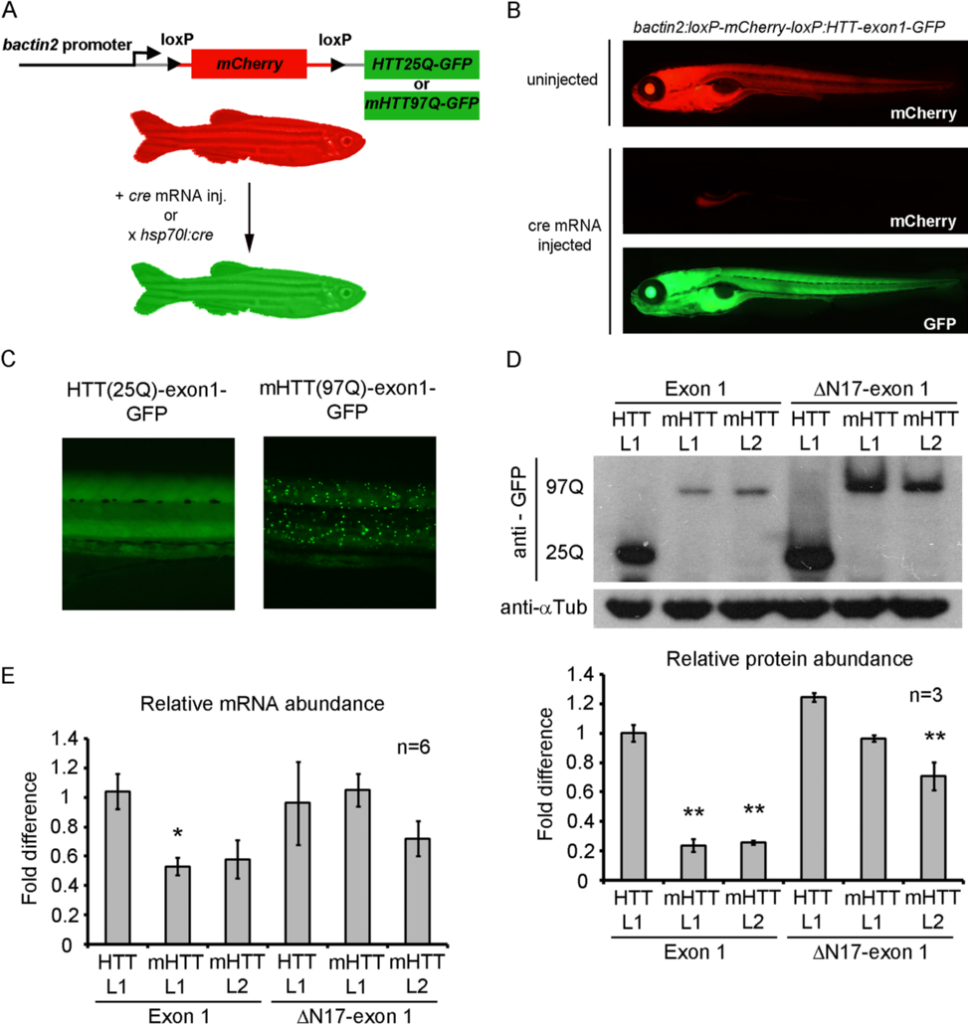
Design and demonstration of Cre-*loxP* inducible HTT-exon 1-GFP expression in transgenic zebrafish. **a** Schematic of the transgene design using the *bactin2* promoter to drive expression of floxed mCherry followed by the HTT-exon 1 cassette being tested. Following Cre expression by mRNA injection or *HS:cre* transgene induction, mCherry is recombined out and the desired HTT-exon 1 cassette fused to GFP is expressed ubiquitously. **b** Photo micrographs depicting 5 day old, HTT-exon1(25Q) embryos displaying mCherry expression when Cre is absent (*upper panel*) or mCherry negative, HTT-exon1-GFP positive expression following *cre* mRNA injection (*bottom two panels*). **c** Close-up view of the trunk of HTT(25Q)-exon1-GFP and mHTT(97Q)-exon1-GFP embryos demonstrating ubiquitous, diffuse expression of HTT-exon1-GFP (*left panel*) and ubiquitous, aggregated expression of mHTT-exon1-GFP (*right panel*). **d** A representative western blot and densitometric measurments for each transgenic line (*n* = 3). Anti-GFP antibody (*top panel*) and anti-tubulin loading controls (*bottom panel*) on protein isolated from pools of 5 day old embryos for each HTT transgenic line crossed to *HS:cre*. 97Q and 25Q mark the location of mHTT-GFP and HTT-GFP protein bands respectively. Protein expression was highest in HTT-exon1 L1 and HTT-ΔN17-exon1 L1 lines. mHTT-exon1 line 1(L1) and line 2(L2) both express at similar levels as do mHTT-ΔN17-exon1 line 1(L1) and line 2(L2). Densitometric measurements (*lower bar graph*) demonstrate significantly lower levels of protein in mHTT-exon1 L1 and L2 compared to all other lines while mHTT-ΔN17-exon1 L2 is significantly lower than HTT-exon1 L1 and HTT-ΔN17-exon1 L1. Comparisons were done by one-way ANOVA with Bonferroni posthoc test, **p < 0.01, error bars are SEM. **e** Quantification of transgene expression by quantitative RT-PCR. Six pools of ten embryos from separate clutches of each HTT transgenic line crossed to *HS:cre* were analyzed. Comparisons of expression level were done by one-way ANOVA with Bonferonni posthoc test, *p < 0.05, error bars are SEM

Since our earlier experiments had demonstrated significant toxicity leading to premature death of transgenic zebrafish upon over-expression of polyQ expanded HTT, we decided to create Cre-*loxP* conditionally inducible transgenic fish. This system allows us to maintain the transgenic lines by bypassing any toxicity by keeping the transgene switched off until expression of Cre recombinase is introduced, which also allows us spatial and temporal control of expression. We chose to use the strong, ubiquitous *bactin2* promoter provided with the tol2kit to drive transgene expression [42]. Each transgene was assembled with the *bactin2* promoter, floxed *mCherry*, followed by one of four HTT-exon1 cassettes (25Q, 97Q(m), ΔN17-25Q, or ΔN17-97Q(m)) fused to GFP flanked by *tol2* transposon sites (Fig. 1a). The polyQ coding DNA sequences were mutated at wobble sites as reported previously [29] to stabilize the transgene and prevent the genomic instability associated with CAG repeats. Transgenic germlines were established for each transgene, one line was maintained for HTT-exon1 and HTT-ΔN17-exon1 control fish while two separate lines were maintained for mHTT-exon1 and mHTT-ΔN17-exon1. Transgenic lines have been maintained to at least F_6_ generation with Mendelian inheritance patterns suggesting a single insertion site is present in each line. Additionally, the use of the Tol2 system should result in single copy transgene insertions. To confirm that these transgenes were inducible by Cre, we injected embryos with Cre mRNA and observed loss of mCherry expression and induction of GFP expression in all lines (Fig. 1b and data not shown). HTT-exon1 L1 and HTT-ΔN17-exon1 L1 lines exhibited ubiquitous, diffuse GFP expression at all time points examined while mHTT-exon1 and mHTT-ΔN17-exon1 lines initially had ubiquitous expression but exhibited GFP^+^ protein aggregates by 5 days post fertilization (dpf), most prominently in trunk muscles (Fig. 1c). To measure the expression level of each transgene in the different lines, western blots and qRT-PCR were performed. Each line was crossed to heat shock inducible Cre (*HS:cre*) transgenic fish and then heat shocked at shield stage resulting in robust, ubiquitous recombination. To evaluate protein levels, Western blots were performed on pools of embryos from three separate clutches harvested at 5 dpf for the HTT-GFP fusion protein and α-tubulin as a loading control (Fig. 1d, upper panel). Densitometry was used to measure relative protein abundance (Fig. 1d, lower panel). Embryos lacking *HS:cre* never show GFP expression (data not shown). HTT-exon1 L1 and HTT-ΔN17-exon1 L1 both exhibit strong GFP expression upon recombination at a size slightly larger than the predicted ~37 kDa and ~35 kDa, suggesting post-translational modification was present. Similarly, both mHTT-exon1 and mHTT-ΔN17-exon1 appear at sizes larger than their predicted ~46 kDa and ~44 kDa molecular masses, which may result from aberrant conformation due to the expanded polyQ and/or post-translational modification (Fig. 1d, upper panel). mHTT-exon1 L1 and L2 exhibit similar levels of expression that are significantly lower than line HTT-exon1 L1, HTT-ΔN17-exon1 L1, and both mHTT-ΔN17-exon1 lines (*p* < 0.01, ANOVA, Bonferroni posthoc test) (Fig. 1d). mHTT-ΔN17-exon1 L1 and L2 also exhibit similar levels of expression that are lower than HTT-ΔN17-exon1 L1 but with only L2 reaching statistical significance (*p* < 0.01, ANOVA, Bonferroni posthoc test) (Fig. 1d). We were surprised to observe the lower protein levels for the mHTT-exon1 lines as compared to the mHTT-ΔN17-exon1 lines since the transgene design and promoter were identical. To test whether transgene mRNA expression was responsible for the difference, we performed qRT-PCR analysis on mRNA extracted from each line. For this analysis, pools of ten GFP^+^ embryos from six independent clutches were assayed for each line. mRNA levels were less varied between lines than protein, with mHTT-exon1 L1 the only one to reach statistical significance in comparison to HTT-exon1 L1 (*p* < 0.05, ANOVA, Bonferroni posthoc test) (Fig. 1e). It is important to note that mRNA levels between mHTT-exon1 lines and mHTT-ΔN17-exon1 lines are similar. This is in contrast to the protein levels, suggesting that post-translational mechanisms may be affecting protein abundance. In fact, the N17 region of HTT has been described as a modifier of protein stability, harboring both phosphorylation [26, 27] and ubiquitination [17] sites that regulate protein degradation. Therefore, it appears that the intact N17 domain leads to greater protein instability in this zebrafish model and its deletion accelerates protein accumulation.

### The transgenic zebrafish expressing mHTT-exon1 lacking the N17 domain exhibits accelerated, progressive movement disorder

**Fig. 2:**
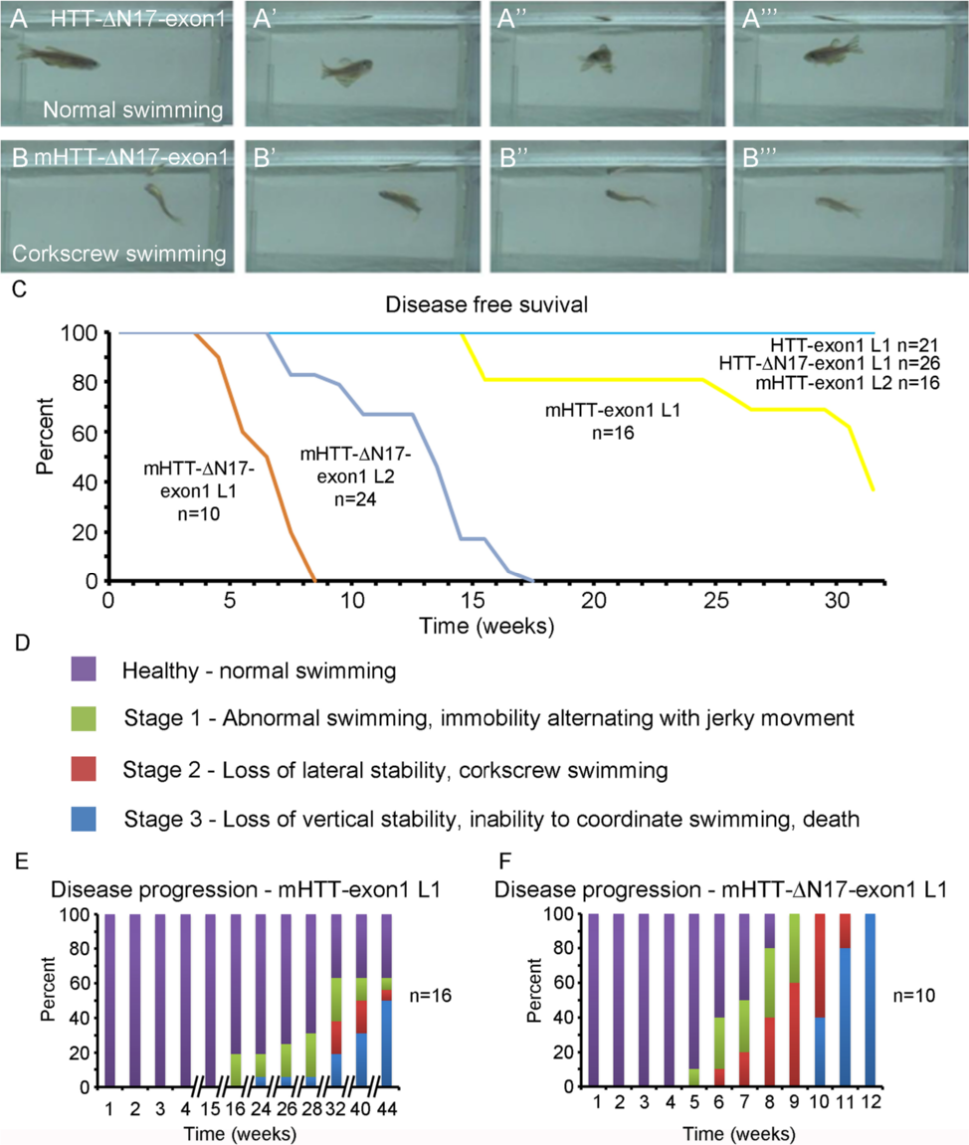
mHTT-∆N17-exon1 transgenic fish develop a progressive motor behavior phenotype. **a** Panels taken from Additional file 1: Movie S1 showing normal swimming behavior in a HTT-ΔN17-exon1 transgenic fish. **b** Panels taken from Additional file 3: Movie S3 showing abnormal swimming behavior, corkscrew swimming, in a mHTT-ΔN17-exon1 transgenic fish. **c** Disease free survival curve for each HTT-exon1 transgenic line corresponding to disease onset. Note that mHTT-ΔN17-exon1 L1 (*n* = 10) and L2 (*n* = 24) both develop symptoms earlier than mHTT-exon1 L1 (*n* = 16) and that mHTT-exon1 L2 (*n* = 16), HTT-exon1 (*n* = 21), and HTT-ΔN17-exon1 (*n* = 26) do not exhibit symptoms in the observed time frame. Kaplan Meier analysis with Log Rank test, *p* < 0.001 for mHTT-ΔN17-exon1 L1, mHTT-ΔN17-exon1 L2, and mHTT-exon1 L1. **d** Behavioral characterization within different transgenic lines. Behavior was grouped into four categories: Healthy, Stage 1, Stage 2, or Stage 3 as described. Observations were made weekly. **e** Disease progression of mHTT-exon1 L1 fish. Note the discontinuous x-axis to account for the extended time frame of behavioral changes (*n* = 18). **f** Disease progression of mHTT-ΔN17-exon1 L1. mHTT-ΔN17-exon1 L1 fish developed a robust, progressive motor behavioral deterioration beginning at 5–8 weeks of age and progressing to immobility and death by 12 weeks (*n* = 10)

Each HTT transgenic line was crossed with *HS:cre* and the embryos heat shocked at shield stage to induce recombination or left as non-heat shocked controls. HTT-exon1 L1 (*n* = 21) and HTT-ΔN17-exon1 L1 (*n* = 26) both exhibited robust GFP expression and developed normally with no behavioral changes up to 26 weeks of age (Fig. 2a and c and Additional file 1: Movie S1 and Additional file 2: Movie S2). mHTT-exon1 L1 (*n* = 16) and L2 (*n* = 16) both survived early development even though mHTT-exon1 protein aggregates were present by 5 dpf. These fish behaved normally until 16 weeks of age, at which point mild behavioral abnormalities, such as abnormal swimming and immobility alternating with jerky movement, were noted in a small number of fish in mHTT-exon1 L1 with more fish displaying these behaviors over time (Fig. 2c). mHTT L2 has not displayed any behavioral abnormalities up to 32 weeks of age. mHTT-ΔN17-exon1 L1 (*n* = 10) and L2 (*n* = 24), in contrast, developed a robust, progressive movement deficit (Fig. 2b and c, and Additional file 3: Movie S3 and Additional file 4: Movie S4). mHTT-exon1, mHTT-ΔN17-exon1 L1, and mHTT-ΔN17-exon1 L2 all exhibit statistically significant differences in survival curve analysis as compared to each other as well as HTT-exon1 L1, HTT-ΔN17-exon1 L1, and mHTT-exon1 L2 (Fig. 2c, Kaplan Meier with Log Rank, *p* < 0.001). We grouped the observed behaviors into three stages according to their severity (Fig. 2d): Stage 1 – abnormal swimming, immobility alternating with jerky movement; Stage 2 – loss of lateral stability, corkscrew swimming (see Fig. 2b); Stage 3 – loss of vertical stability, inability to coordinate swimming, death. mHTT-exon1 L1 fish begin exhibiting symptoms at 16 weeks and slowly progress so that by 44 weeks 50 % of the fish have reached Stage 3 (Fig. 2e). However, 40 % of the fish are still unaffected. mHTT-ΔN17-exon1 L1 begins exhibiting Stage 1 symptoms by 5 weeks of age and progresses to Stage 3 by 10–12 weeks (Fig. 2f). mHTT-ΔN17-exon1 97Q L2 does not exhibit Stage 1 symptoms until 8 weeks and progresses more slowly with Stage 3 not reached for all fish until after 20 weeks (Additional file 5). These experiments have been repeated in multiple generations with similar results. Non-heat shocked control transgenic fish for all lines, including both mHTT-ΔN17-exon1 lines, develop normally and do not show abnormal movement before 1 year of age (the last observation made), although some leaking recombination was noted. Overall, we have found that mHTT-ΔN17-exon1 transgenic fish consistently exhibit a progressive movement deficit, while mHTT-exon1 fish show a later-onset and more slowly progressing behavioral impairment. Importantly, the control HTT-exon1 and HTT-ΔN17-exon1 fish are indistinguishable from wild-type fish. Since the phenotypes of the transgenic fish were analyzed up to 11 months of age, it is possible that the mHTT-exon1 zebrafish could develop more severe disease-like phenotypes as they age.

### The transgenic zebrafish expressing mHTT-ΔN17-exon1 exhibit reduced brain weight

**Fig. 3:**
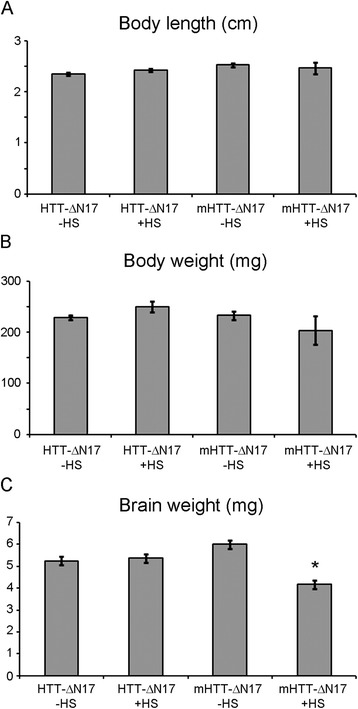
mHTT-ΔN17-exon1 transgenic fish exhibit reduced brain weight. **a** Body length is not statistically different between HTT-ΔN17-exon1 and mHTT-ΔN17-exon1 without (−HS) or with heat shock (+HS) (*n* = 8 for each condition). **b** Body weight is not statistically different between HTT-ΔN17-exon1 and mHTT-ΔN17-exon1 with or without heat shock (*n* = 8 for each condition). **c** The brain weight of mHTT-ΔN17-exon1 after heat shock induction of the HTT transgene is significantly less than either non-heat shocked mHTT-ΔN17-exon1 fish or heat shocked HTT-ΔN17-exon1 fish (*n* = 8 for each condition). (*) *p* < 0.05 by Student’s *t*-test. Error bars are SEM

To examine if brain atrophy, a hallmark of neuropathology in HD, was present in the mHTT-ΔN17-exon1 fish, brain weight from Stage 3 fish was compared to non-heat shocked fish and control HTT-ΔN17-exon1 with and without heat shock. Fish from each group were age matched and raised under equal population density and tank size. This is an important consideration for zebrafish since they exhibit density dependent growth [43]. The average body length and weight of each group of fish was not significantly different from each other (*n* = 8 for each group) (Fig. 3, a and b). The zebrafish brains from each line (excluding the olfactory bulbs and eyes) were dissected and weighed. Interestingly, heat shocked, Stage 3 mHTT-ΔN17-exon1 fish exhibited a significantly smaller brain weight than the brains from non-heat shocked mHTT-ΔN17-exon1 fish or HTT-ΔN17-exon1 fish with or without heat shock (Fig. 3c) (ANOVA with Bonferroni post hoc, *p* < 0.05). This result suggests that mHTT-ΔN17-exon1 fish exhibit brain atrophy similar to that seen in HD patients.

### N17 domain determines the cytoplasmic versus nuclear accumulation of mutant HTT exon1 fragments in vivo

**Fig. 4:**
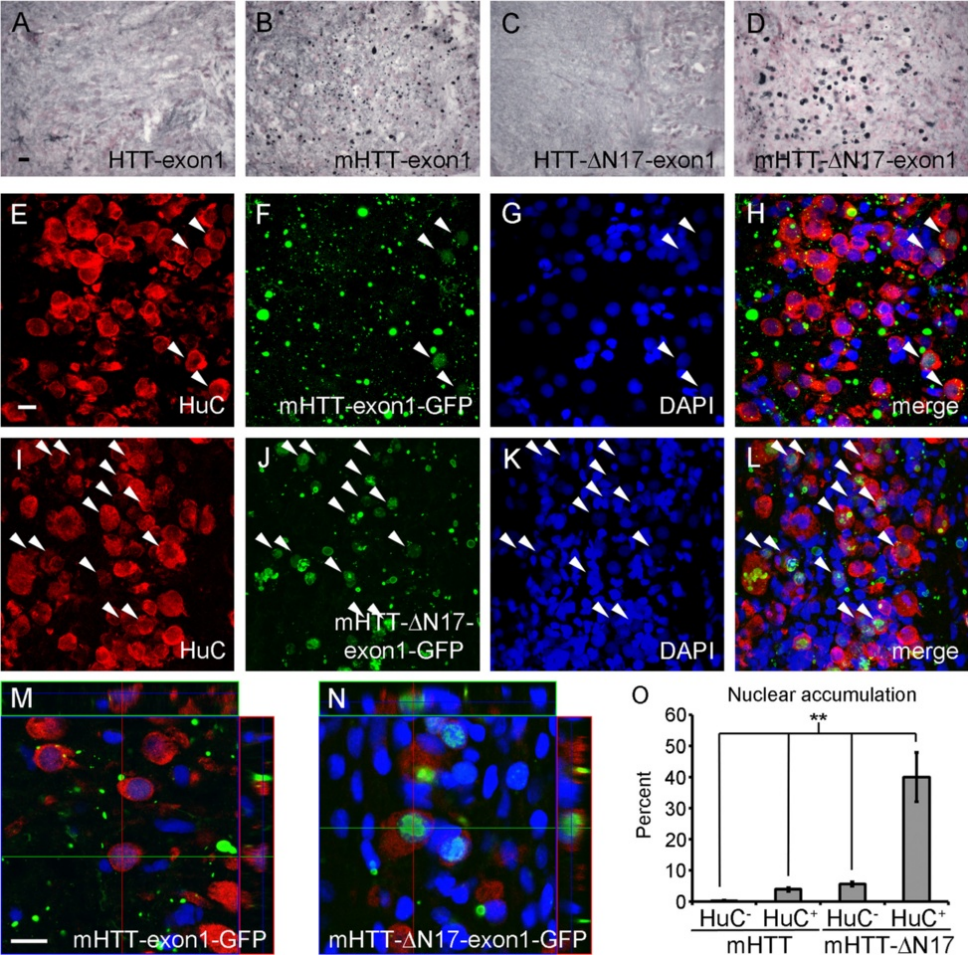
mHTT-ΔN17-exon1 accumulates mainly in the nucleus of neurons while mHTT-exon1 aggregates are mostly outside the neuronal cell body. Brain sections from 26 week old HTT-exon1 and mHTT-exon1 fish or 12 week old HTT-ΔN17-exon1 and mHTT-ΔN17-exon1, Stage 3 fish were immunostained to observe the transgenic HTT-exon1 localization. All images are from the hindbrain region and similar staining was present throughout all brain regions in each fish. **a**–**d** S830 anti-human HTT Exon 1 antibody was used to detect transgene expression in each transgenic line. HTT-exon1 (**a**) and HTT-ΔN17-exon1 (**c**) tissue exhibits uniform, ubiquitous expression. mHTT-exon1 (**b**) tissue displays many small but distinct aggregates while mHTT-ΔN17-exon1 (**d**) tissue has many large aggregates. Scale bar equals 25 μm. **e**–**l** Immunofluorescent staining of mHTT-exon1 (**e**–**h**) and mHTT-ΔN17-exon1 (**i**–**l**) transgenic fish for neurons (HuC), transgenic HTT-exon1-GFP fusion protein, and nuclei (DAPI). mHTT-exon1 tissue exhibits many small GFP^+^ aggregates that are not generally associated with HuC positive neuronal cell bodies suggesting they are either in the axons or dendrites or are non-neuronal (**e**–**h**). Occasional, weak nuclear accumulation is present in a few neurons (*white arrowheads*). mHTT-ΔN17-exon1 tissue has many neuronal, HuC positive, nuclei co-localized with HTT-exon1-GFP (**i**–**l**). Scale bar equals 10 μm. **m** and **n** 3D confocal projections of mHTT-exon1 (**m**) and mHTT-ΔN17-exon1 (**n**) tissue demonstrating weak nuclear HTT-exon1-GFP staining in mHTT-exon1 and strong nuclear staining in mHTT-ΔN17-exon1. Scale bar equals 10 μm. **o** Quantification of protein accumulation location. Nuclear HTT-exon1 aggregates were rare in HuC negative cells of both lines and mHTT-exon1 transgenic HuC positive neurons. mHTT-ΔN17-exon1 transgenic fish exhibited robust nuclear accumulation of HTT-exon1-GFP in ~40 % of HuC positive cells. ANOVA with Bonferroni posthoc test, (**) *p* < 0.01

The 17N-terminal amino acids of HTT have been reported to regulate protein stability, toxicity, and sub-cellular localization. In a mouse BACHD model with N17 deleted in the context of the full length *HTT* gene, dramatically accelerated and exclusive nuclear accumulation of mHTT fragments were demonstrated [28]. In the context of mHTT-exon1, an HD-relevant pathogenic fragment [32], N17 is necessary for nuclear export/cytoplasmic targeting in vitro [44, 45]: its relevance in mediating nuclear versus cytoplasmic pathology in vivo remains unclear. Therefore, we assessed if the localization of aggregates was different in transgenic HD fish expressing mHTT-exon1 with or without intact N17. First, we immunostained brain sections of Stage 3, *HS:cre*/mHTT-ΔN17-exon1 fish, *HS:cre*/HTT-ΔN17-exon1 fish, *HS:cre*/mHTT-exon1 fish, and *HS:cre*/HTT-exon1 fish with the anti-human HTT antibody S830 [46] (Fig. 4a–d). HTT-ΔN17-exon1 and HTT fish exhibit diffuse staining throughout the brain with no visible aggregates (Fig. 4a and c). mHTT-ΔN17-exon1 and mHTT-exon1 fish both exhibit robust aggregate staining throughout the brain (Fig. 4b and d). The aggregates in mHTT-exon1 appear as small puncta with occasional larger accumulations (Fig. 4b) while the aggregates in mHTT-ΔN17-exon1 appear mainly as large oval shaped staining reminiscent of nuclei (Fig. 4d).

To examine the cellular and subcellular localization of these aggregates in more detail, we combined immunofluorescent staining for neurons (anti-HuC) with GFP staining of the HTT-exon1 fusion protein and DAPI for nuclei. We focused our examination on the ventrolateral nucleus of torus semicircularis, a brain region with strong HuC staining and a mixture of cell bodies and neuropil. In HTT-exon1 and HTT-ΔN17-exon1 fish, diffuse GFP staining was present throughout the brain including neurons (Additional file 6). Close examination by confocal microscopy demonstrated that HTT-exon1 is largely excluded from the nucleus while HTT-ΔN17-exon1 is detectible in the nucleus suggesting the nuclear export function of N17 is intact in vivo (Additional file 6). mHTT-exon1 fish exhibited robust aggregate formation, with the majority localized away from HuC positive neuronal cell bodies (Fig. 4e–h). These aggregates are likely present in the neuropil (e.g. axons and dendrites), but it is also possible that a subset of them are present in non-neuronal cells. Occasional GFP^+^ aggregates are present in the peri-nuclear regions stained by HuC or weakly and diffusely in DAPI positive nuclei (Fig. 4e–h, arrowheads highlight GFP^+^ nuclei). mHTT-ΔN17-exon1 fish, on the other hand, exhibited strong and predominant nuclear accumulation of mHTT-ΔN17-exon1 that was found almost exclusively in the HuC positive neurons (Fig. 4i–l). Neuropil aggregates similar to those seen in mHTT-exon1 fish were also present, but were much less abundant. 3D confocal projections clearly demonstrated the neuropil localization of HTT aggregates in mHTT-exon1 fish (Fig. 4m) and nuclear accumulation in the mHTT-ΔN17-exon1 fish (Fig. 4n). Quantification of HTT-GFP location demonstrates significant enrichment of nuclear mHTT-∆N17-exon1 in HuC positive neurons of mHTT-ΔN17-exon1 fish (Fig. 4o, *p* < 0.05, ANOVA with Bonferonni post hoc). No difference was detected in cell density, percent HuC positive cells, or aggregate density (nuclear accumulation was scored as a single aggregate) in mHTT-exon1 versus mHTT-ΔN17-exon1 fish (Additional file 7). Taken together, our study is consistent with the in vitro studies in cells and mouse study with BACHD-∆N17 mice that N17 is a crucial element in preventing nuclear pathogenesis for mHTT-exon1, and our study provides the first in vivo evidence that N17 can prevent nuclear accumulation and aggregation of a known pathogenic mHTT fragment, mHTT-exon1, in a vertebrate model of HD.

### Mutant HTT exon1 lacking the N17 domain exerts toxicity selectively in neurons to induce movement deficits in HD transgenic zebrafish

**Fig. 5:**
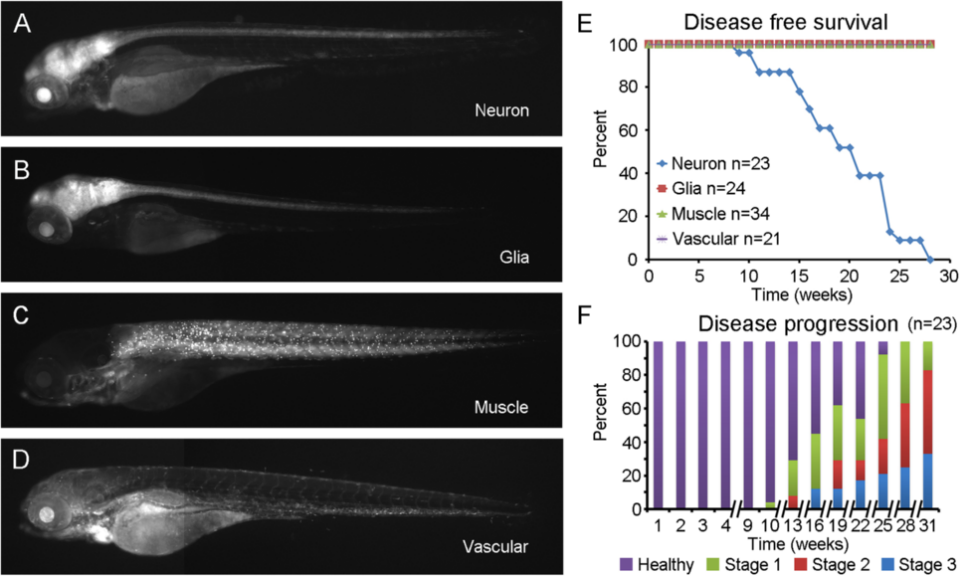
Neuron specific activation of mHTT-ΔN17-exon1 causes motor behavioral phenotype. **a** *elavl3:cre* (*n* = 23), **b** *gfap:cre* (*n* = 24), **c** *mylpfa:cre* (*n* = 34), and **d** *etv2:cre* (*n* = 21) transgenic lines were incrossed to mHTT-ΔN17-exon1 to activate the transgene specifically in neurons, glia, muscle, and vascular cell lineages respectively. GFP fluorescence in living embryos demonstrates the spatially restricted expression of each line at 5 days post fertilization. **e** A disease free survival curve demonstrating that only *elavl3:cre*/mHTT-ΔN17-exon1 fish develop a behavioral phenotype similar to ubiquitous activation of mHTT-ΔN17-exon1, *p* < 0.01, Kaplan Meier survival analysis. **f** Disease progression of *elavl3:cre*/mHTT-ΔN17-exon1 fish is similar to ubiquitious mHTT-ΔN17-exon1 fish although temporally delayed, note the discontinuous x-axis (*n* = 23). Behavioral Stages are described in Fig. 2

HD is generally thought to be caused by neuron specific dysfunction and atrophy. However, since HTT expression is ubiquitous it is possible that disease specific pathologies are occurring in other cell types. HTT-polyQ expression in glia [47], immune cells [48], skeletal muscle [49] and vasculature [50, 51] have all been suggested to contribute to HD. A recent study using conditional BAC transgenic mouse models of HD (BACHD) revealed distinct and synergistic roles of full-length mHTT expressed in cortical pyramidal neurons and striatal MSNs in eliciting multiple behavioral deficits and selective neurodegeneration [52]. However, these prior models lack the overt and progressive movement disorder seen in our mHTT-ΔN17-exon1 fish model, and it is unclear if mHTT-exon1 lacking N17 is ubiquitously toxic or may retain pathogenic specificity similar to the intact mHTT-exon1. To begin answering such a question, we crossed mHTT-ΔN17-exon1 fish with Cre driver lines with selective Cre expression in lineages for neurons (*elavl3-Cre*) [53], glia (*gfap-Cre*) [54], skeletal muscle (*mylpfa-Cre*) [55], and endothelial (*etv2-Cre*) [56] cells. Each cross gave expected tissue and cell-type specific mHTT-exon1 transgene expression (Fig. 5a–d). GFP^+^ fish from each cross were selected and raised for behavioral observation (*n* = >20 for each genotype). Only the neuron specific, *elavl3:cre*/mHTT-ΔN17-exon1 fish developed movement abnormalities reminiscent of the ubiquitously activated mHTT-ΔN17-exon1 (Fig. 5e, *p* < 0.001, Kaplan Meier analysis). Using our previously described behavioral categories, we found that *elavl3:cre*/mHTT-ΔN17-exon1 fish exhibit all of the progressively deteriorating movement behaviors described in the ubiquitously expressing fish (Fig. 5f). It should be noted that the movement abnormalities observed in *elavl3:cre*/mHTT-ΔN17-exon1 fish appeared and progressed more slowly than ubiquitously expressing fish, suggesting the possibility that non-neuronal cells modify the effect of mHTT on neurons in this model.

**Fig. 6:**
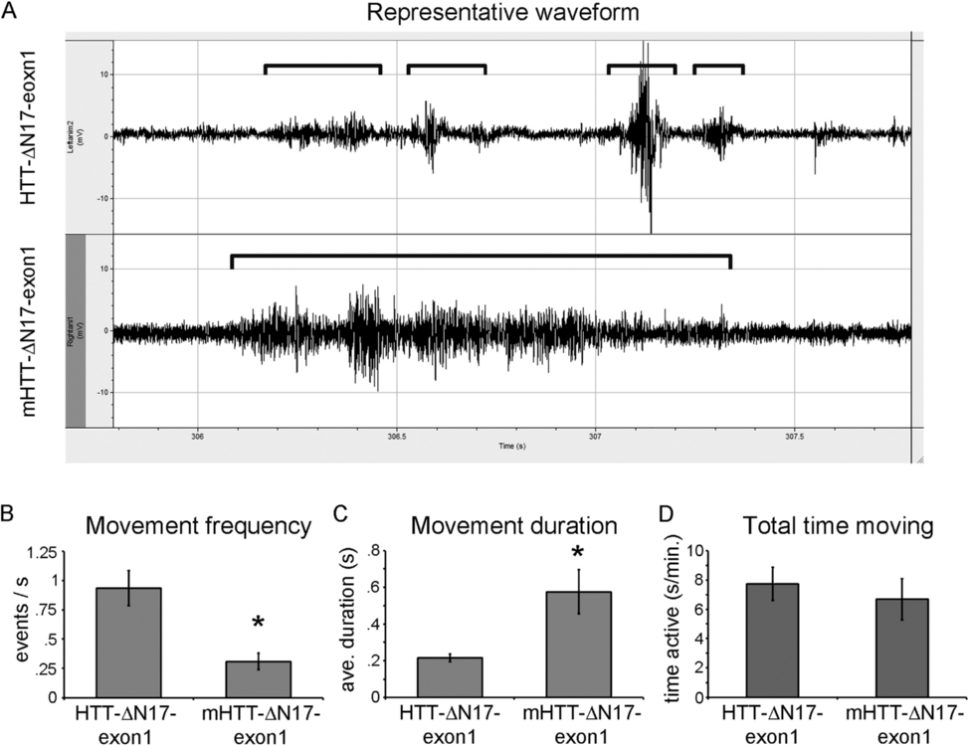
Behavioral characterization of *elavl3:cre*/mHTT-ΔN17-exon1. Field potential recordings were made from freely behaving *elavl3:cre*/HTT-ΔN17 fish (*n* = 6) and *elavl3:cre*/mHTT-ΔN17-exon1 (*n* = 6) fish exhibiting Stage 1 behaviors. Field potentials represent the electrical muscle activity of a given freely behaving animal as measured by recording electrodes placed in the chamber during movements. Each fish was allowed to acclimate to the recording chamber for 5 min and then recorded for 5 min. **a** Representative field potential recordings from an *elavl3:cre*/HTT-ΔN17-exon1 fish (*top*) and an *elavl3:cre*/mHTT-ΔN17-exon1 fish (*bottom*). *Brackets* define of swimming activity. Note the short, distinct swimming activity of the *elavl3:cre*/HTT-ΔN17-exon1 fish versus the sustained activity of the *elavl3:cre*/mHTT-ΔN17-exon1 fish. **b** Average movement frequency as calculated by counting each distinct, non-overlapping movement waveform over time. *elavl3:cre*/mHTT-ΔN17-exon1 fish move significantly less often than *elavl3:cre*/HTT-ΔN17-exon1 fish. **c** *elavl3:cre*/mHTT-ΔN17-exon1 fish display extended periods of activity as compared to *elavl3:cre*/HTT-ΔN17-exon1 fish. **d** Total time moving is not significantly different between *elavl3:cre*/HTT-ΔN17-exon1 and *elavl3:cre*/mHTT-ΔN17-exon1 fish. (*) *p* < 0.05 by Student’s *t*-test. Error bars are SEM

To examine the behavior of these fish in a more quantitative manner, we performed field potential recordings [57] of freely behaving *elavl3:cre*/mHTT-ΔN17-exon1 (*n* = 6) and *elavl3:cre*/HTTΔN17 fish (*n* = 6) fish (Fig. 6). *elavl3:cre*/mHTT-ΔN17-exon1 fish were categorized as Stage 1 at the time of this experiment while *elavl3:cre*/HTT-ΔN17-exon1 fish were categorized as healthy. Field potential recordings are performed by placing individual fish into a small chamber with recording electrodes at each end. When a fish moves the electrical activity of the muscle creates a potential across the electrodes that can be recorded over time. This gives an indirect measurement of each fish’s motor activity. *elavl3:cre*/HTT-ΔN17-exon1 fish exhibit short bursts of activity corresponding to swimming and turning movements within the chamber (Fig. 6a, top panel). *elavl3:cre*/mHTT-ΔN17-exon1 fish exhibited bouts of prolonged activity (Fig. 6a bottom panel and c). However, *elavl3:cre*/mHTT-ΔN17-exon1 fish initiated movements less frequently than *elavl3:cre*/HTT-ΔN17-exon1 fish (Fig. 6b). Overall, *elavl3:cre*/HTT-ΔN17-exon1 and *elavl3:cre*/mHTT-ΔN17-exon1 fish did not differ in the total amount of time they were active during these trials (Fig. 6d). These results support the behavioral observations that Stage 1 fish exhibit bouts of jerky movement followed by inactivity.

In summary, our cell-type-specific expression of mHTT-ΔN17-exon1 revealed a remarkable specificity of toxic mHTT fragments to neurons while its toxicity to several non-neuronal cell types including glia, muscle and blood vessels are unremarkable. This result suggests the neurons are particularly sensitive to the nuclear polyQ toxicity in the context of mHTT-exon1, and the presence of N17 domain can delay such toxicity in vivo.

## Discussion

The molecular mechanisms causing HD have been difficult to identify even though the specific gene mutation has been known for 20 years. Many research models have been created in diverse organisms such as rats [58], mice [29, 33, 59] zebrafish [39, 40], fly [37], worm [35, 36], and yeast [34]. Mammalian models have been extremely valuable in validating disease mechanisms and testing candidate therapeutics [30]; they also have limitations such as lack of overt disease phenotypes and limited scalability due to the cost and effort to maintain a large rodent colony. Invertebrate models are highly scalable but lack close genetic homology to humans. The zebrafish model is an ideal compromise between the scalability of invertebrate models and genetic homology to vertebrates. Previously developed zebrafish models of HD are not ideal models of the disease due to lethality caused by high level overexpression of toxic mHTT fragments in embryos or limited disease relevance due to restricted tissue expression.

Here we present a novel Cre-*loxP* inducible zebrafish model of HD. This model allows for precise spatial and temporal control of expression of the disease linked protein, which was previously only available in the mouse [52, 60]. These transgenic lines can be maintained in non-recombined form and experimental clutches of embryos generated as needed by Cre mRNA injection or intercross with Cre driver transgenic lines. By incrossing tissue specific Cre driver lines to the mHTT-ΔN17-exon1 line we found that neurons appear to be the main cell involved in the abnormal movement behaviors originally observed with ubiquitous expression of this particular toxic polyQ protein. This is in agreement with findings in conditional mouse HD models [52, 60, 61]. Future work will be necessary to establish the specific neuronal population responsible for the behavioral phenotype in this zebrafish model using neuronal sub-type-specific Cre driver lines. Additionally, careful cell-type specific analysis for histological and molecular markers of HD-like pathologies will be necessary to further define the similarities to human disease.

These transgenic fish are based upon expression of mHTT-exon1. In recent years it has become apparent that the exon 1 fragment of the much larger HTT protein not only contains the expanded polyQ region that is mutated in the disease but is also a known pathogenic species in the diseased as a result of aberrant mHTT splicing event [32] and proteolysis [31]. A surprising finding in our study is that zebrafish is somewhat refractory to the toxicity of ubiquitously expressed mHTT-exon1, since only a few transgenic mHTT-exon1 fish developed abnormal movements by 6 months of age. Histological examination of these fish demonstrated robust mHTT-exon1-GFP positive protein aggregates formed in the brain, and most of these aggregates are located in the neuropil (e.g. axons, dendrites). One leading hypothesis is that the nucleus is the site of pathological action of expanded polyQ in all polyQ disorders [4]. It has been reported that the N17 terminal amino acids of HTT contains a nuclear export signal [45, 62]. Additionally, these amino acids have been reported to function in modifying aggregate formation and protein stability [17, 23, 24, 26, 27]. Recently, Gu et. al. [28] showed that deletion of these 17 amino acids in the context of BAC transgenic mice harboring the full length human HTT gene with 97Q causes an adult-onset progressive movement disorder and robust striatal neurodegeneration. Interestingly, these BACHD-∆N17 mice also presents with dramatic acceleration of selective nuclear mHTT aggregates due to accumulation of small mHTT polyQ fragments. We therefore, tested whether deletion of these 17 amino acids in the context of an HTT exon 1 based transgene would cause accelerated disease in our zebrafish model. Mutant HTT-ΔN17-exon1 expression caused a rapidly developing progressive movement disorder that ultimately led to early death. The development of this behavioral phenotype was correlated with massive accumulation of mHTT-∆N17-exon1-GFP in the nucleus of neurons. Interestingly, although expression was ubiquitous, large nuclear aggregates were only observed in neurons, suggesting mechanisms of nuclear accumulation and aggregation of the mutant polyQ protein is neuronal selective. We did not observe robust nuclear accumulation of the non-toxic HTT-ΔN17-exon1 transgene suggesting that lack of N17 function alone without polyQ expansion is not sufficient to elicit high levels of nuclear protein accumulation. Our study confirmed and extended the results found in BACHD-∆N17 mice by demonstrating not only that N17 is crucial in preventing nuclear mHTT toxicity and onset of disease-like phenotypes in an vertebrate, but also provide strong evidence that N17 is crucial in mitigating the ability of mHTT-exon1 fragment, a known pathogenic species in HD, to mediate its nuclear toxicity and disease pathogenesis in an intact vertebrate model.

A truly surprising finding in our study is that the mHTT-∆N17-exon1 fragment elicits robust and progressive movement disorder through its toxicity in neurons, but not in astrocytes, skeletal muscles, or vascular cells. This result showed that neurons, unlike the non-neuornal cells, may have selective impairment in managing the proteostasis of the expanded polyQ proteins, especially when such protein is translocated and accumulated in the nucleus. Our result is consistent with the rich evidence that expanded polyQ protein is neurotoxic in an artificial context of an endogenous protein [63], or other polyQ diseases (e.g. SBMA or SCA1) in which nuclear translocation is essential to disease pathogenesis [4]. Our study has important implications in the context of understand HD pathogenesis and therapy. First, it suggests that despite the fact that mHTT-exon1 is naturally occurring and is more toxic than longer mHTT-exon1 fragments or full-legnth mHTT, the N17 domain can still provide substantial protection against its ability to translocate into the nucleus and elicit severe disease. Second, since in HD patients and HD mice mHTT fragments (including exon1 fragment) eventually accumulate in the nucleus and cause nuclear pathoglogy [10, 31], the mHTT-ΔN17-exon1 zebrafish represent a feasible model of HD. Thus, better understanding how N17 function is compromised in the context of HD neurons or how to improve the ability of the nucleus to clear such aberrant polyQ proteins may be important steps in reducing disease burden in HD.

Finally, our study may have important implications in the use of zebrafish models to study pathogenesis or testing candidate therapeutics for HD. An important advance we have made, through the use of conditional genetics in zebrafish, is the creation of a stable and robust model of HD with progressive movement deficits and brain pathology. The inducibility of the model allows us to study the toxicity of mutant polyQ protein in distinct cell populations. Since the model recapitulates aspects of the disease related to nuclear accumulation of mHTT-polyQ fragments, our model may be particularly useful to study mechanism and therapeutics aiming to improve nuclear proteostasis or reduce the consequence of nuclear mHTT toxcitiy in vivo. Moreover, due to the robust brain atrophy associated with the overt and progressive movement disorder, our model could also be used to test novel neuroprotective therapies. Given the obvious advantage of the zebrafish system in terms of its scalability and relative low cost in its maintenance, we envision that the mHTT-ΔN17-exon1 fish model will facilitate the screening of potential genetic disease modifiers or candidate therapeutics at a scale that is not likely to be achievable in the other vertebrate models of HD.

## Methods

### Animal husbandry

All zebrafish use was approved by the University of California, Los Angeles Animal Care and Use Committee. This work was performed under IACUC protocol ARC # 2001-074-41 issued by OFFICE OF ANIMAL RESEARCH OVERSIGHT, University of California Los Angeles. Adults, juveniles, and zebrafish embryos were maintained according to standard zebrafish methods [64]. Heat shock was performed on shield stage embryos for 30 min at 38.5 °C after which embryos were transferred to a standard zebrafish incubator at 28.5 °C to be raised.

### Transgene constructs and generation of transgenic zebrafish

All transgenes were generated using the Multisite Gateway System (Life Technologies) and the Tol2kit developed for zebrafish [42]. p3E-HTT Exon 1 25Q-EGFP, p3E-HTT Exon 1 97Q-EGFP, p3E-ΔN17 HTT Exon 1 25Q-EGFP, and p3E − ΔN17 HTT Exon 1 97Q-EGFP were generated by BP reaction of each respective PCR product flanked with B2F and B3R sequences to pDONR™ P2R-P3. Transgene plasmids were created by LR multisite reaction of pDESTTol2pA, p5E-*bactin2*, pME-loxPmCherryloxP, and each respective HTT p3E vector. Resulting plasmids were injected into single cell zebrafish embryos along with Tol2 mRNA using standard methods to efficiently generate germline integrations. Resulting F_0_ fish were raised and their offspring screened for ubiquitous mCherry expression. Positive F_1_ embryos were raised to establish each line. Each line was derived from separate F_0_ fish to control for integration effects.

Cre driver lines were generated by LR multisite reaction with pDESTTol2pA, promoter specific p5E vector, pME-Cre, and p3E-pA. p5E-*elav3l* was generated by cloning the 3.1 kilobase zebrafish *elav3l* (also known as *HuC*) promoter, into pDONR™ P4R-P1R using the BP reaction. Similarly p5E-*gfap* was generated by subcloning the *gfap* promoter from the Tg(*gfap*:GFP) plasmid [54] into pDONR™ P4R-P1R. p5E-*mylpfa* [65] and p5E-*etv2* [56] were previously reported. Each transgenic line was generated as described above. However since these lines do not have a fluorescent marker gene, founders were identified by outcross to *ubi:Switch* fish [66] which exhibit a GFP to mCherry fluorescent switch in tissues expressing Cre recombinase. Independent lines with strong tissue specific recombination and minimal leakiness were maintained for each promoter.

### qRT-PCR

Quantitative RT-PCR was performed on cDNA generated using Superscript III Reverse Transcriptase (Life Technologies, 18080044) from total RNA extracted from 5 day post fertilization larvae using Trizol reagent (Life Technologies, 15596026) on a Stratagene Mx3005P qPCR system. Three pools of ten embryos from separate clutches of each transgenic line were analyzed. Oligo(dT)12-18 (Life Technologies, 18418–012) was used to prime the reverse transcriptase reaction. Primers were: βactin1F, TGTTTTCCCCTCCATTGTTG; βactin1R, ACATACATGGCAGGGGTGTT; EGFPqF, ACGTAAACGGCCACAAGTTC; EGFPqR, AAGTCGTGCTGCTTCATGTG.

### Western blotting

Western blots were performed on protein extracted from 5 day post fertilization embryos using standard methods. Five embryos were pooled for each sample into 25 μl of 2X SDS Loading Buffer. Protein samples were run on a 12 % Tris-glycine acrylamide gel (Thermo Scientific, 25247). Protein was transferred onto a PDVF membrane (Thermo Scientific, 88520), and then probed with rabbit anti-GFP primary antibody 1:2000 dilution (Life Technologies, A11122), stripped and then reprobed with mouse anti-tubulin antibody 1:10,000 dilution (Sigma, T5168). Secondary antibodies were anti-rabbit-peroxidase (Sigma, A0545) and anti-mouse-peroxidase (Sigma, A9044) both at 1:5000 dilutions. Chemiluminescent detection was performed using Lumi-Light western blotting reagent (Roche, 12015200001) and HyBlot CL autoradiography film (Denville Scientific, E3018). Film was digitized on an HP Scanjet G3110 and the image adjusted in Adobe Photoshop CS4. ImageJ was used to perform densitometric measurements.

### Behavioral analysis

Fish were raised at similar densities and monitored weekly for abnormal movement. Fish were grouped into four categories: 1) Healthy – normal swimming; 2) Stage 1 – abnormal swimming, immobility alternating with jerky movements; 3) Stage 2 – loss of lateral stability, corkscrew swimming; 4) Stage 3 – loss of vertical stability, inability to coordinate movement, death. When fish were no longer able to swim for feeding they were killed for further analysis.

For more detailed behavioral analysis we performed electric field potential recordings on freely behaving adult *elav3l:cre*/HTT-ΔN17 or, Stage 1 categorized, *elav3l:cre*/mHTT-ΔN17 fish (*n* = 6 each) according to the methods of Issa et.al. [57]. Fish were placed into a recording chamber and allowed to acclimate to the chamber for 5 min. Then field potentials generated from muscles during movement were recorded for 5 min for each fish. The recording chamber was 11 cm long × 4 cm wide × 3 cm deep containing double distilled water with a resistance of ~ 15 MΩ. Water depth was 2.75 cm. Room temperature was set to 25 °C. Electric field potentials were recorded using one pair of electrodes (0.5 cm exposure, 0.1 cm thick of bare copper medal) placed on either end of each testing chamber. Electric signals were amplified 1000-fold using an AC differential amplifier (AM-Systems model 1700, Carlsborg, WA USA), low-pass filtered at 300 Hz and high-pass filtered at 500 Hz. All signals were digitized and stored with a Digidata-1322A and acquired using Axoscope software (Molecular Devices, Inc., Sunnyvale, CA, USA). Data was annotated manually and movement frequency, duration, and total time were calculated.

### Body length, weight, and brain weight measurements

Body length and weight were measured on fish deeply anesthetized with Tricaine solution (Sigma, A5040). Length was measured from the tip of the jaw to the base of the tail since the *HS:cre* line was in a long-fin genetic background resulting in mixed normal and long-fin offspring. After measuring the body, fish were killed by exsanguination, the top of the skull removed, and the head fixed in 4 % paraformaldehyde overnight at 4 °C to improve tissue stability for dissection. Fish heads were washed three times in PBS and gently dried. Brains were carefully dissected using watchmakers forceps from the olfactory bulb-telencephalon junction to the hindbrain-spinal cord junction. All cranial nerves were carefully removed except for the optic nerve and optic tract which remained attached to the brain. The eyes were removed. Excess liquid was removed and brain weight was measured.

### Histology and immunostaining

To harvest tissue for cryo-sectioning, adult fish were deeply anesthetized in Tricaine solution (Sigma, A5040) and then killed by exsanguination. The head was removed as was the top of the skull. Fish heads were fixed overnight in 4 % paraformaldahyde/PBS solution at 4 °C with constant movement on a nutator mixer. The following day the brain was dissected out intact from the tip of the forebrain to the hindbrain spinal cord junction. The brain was then fixed overnight in 4 % paraformaldahyde/PBS solution plus 5 % sucrose at 4 °C with constant movement on a nutator mixer. The following day fixed tissue was put through an increasing sucrose gradient: 5, 10, 12.5, 15, and 20 % in PB buffer, 1 h each and then placed at 4 °C with constant movement on a nutator mixer overnight. The following day tissue was infiltrated with a mix of 2:1 20 % sucrose solution to OCT Compound (Tissue-Tek, 4583) for 1 h and then snap frozen in 100 % OCT in aluminum foil molds placed into a 2-methylbutane (Sigma, M32631), dry ice bath. Blocks were stored at −80 °C until they were cut at 16 μm for immunostaining on a Leica model CM3050S cryostat.

Immunohistochemistry was performed on cryosections using the polyclonal sheep anti-human HTT primary antibody S830 [46] at 1:1000 dilution. Biotinylated anti-sheep secondary (Vector Laboratories, BA-6000) was used at 1:200 dilution and the Vectastain ABC Elite kit (Vector Laboratories, PK-6100) plus Vector SG substrate (Vector Laboratories, SK-4700) used to perform the color reaction. Sections were then counter stained with Nuclear Red (Vector Laboratories, H-3403) and mounted with Permount (Fisher, SP15-100).

Immunofluorescence was performed on sections using manufacturer’s recommended protocol and dilution for each antibody. Primary antibodies were rabbit anti-GFP (Life Technologies, A11122), 1/200 dilution, and anti-HuC/HuD (Life Technologies, A21271), 5 μg/mL. Secondary antibodies were goat anti-rabbit Alexa488 IgG (Life Technologies, A11008), 1/200 dilution, and donkey anti-mouse Rhodamine (Life Technologies, A16016), 1/200 dilution. Stained sections were mounted in Prolong Gold Antifade with DAPI (Life Technologies, P36935).

### Imaging

Images were captured on an Axioskop 2 plus microscope (Zeiss) using 5×, 10×, or 40× objectives with a Hamamatsu ORCA-ER camera and Volocity 5 software (Improvision). Confocal images were collected on a Zeiss LSM 510 with a 63× oil immersion objective (NA 1.4). Adobe Photoshop CS4 was used to adjust brightness and contrast and assemble composite images.

To quantify GFP^+^ protein aggregates in HuC stained sections, ImageJ was used to count cell number (DAPI positive nuclei), HuC^+^ cells, and aggregate number. Maximum intensity projections of 133.6 μm × 133.6 μm × 4.7 μm z-stacks were analyzed from three non-consecutive sections of each fish. Images were acquired such that bright GFP+ aggregates did not over saturate the image. Therefore, the background level of GFP expression appears lower than it actually was. Three fish were examined for each genotype.

### Statistical analsysis

Student’s *t-test* was used for single comparisons, ANOVA with Bonferonni posthoc test was used for multiple comparisons, and Kaplan Meier survival analysis with Log Rank posthoc test was used for survival curve comparisons with *p* < 0.05 set as significant for each. All statistical analysis was performed using SPSS 14.0 software.

## Conclusions

We have developed a Cre-inducible HD model in zebrafish base upon expression of human exon 1 of *HTT*. Using this model we have demonstrated that the N17 domain is protective in the context of 97Q since its deletion results in more severe phenotypes and earlier lethality. Deletion of N17 results in neuron-specific accumulation of 97Q in the nucleus and neuron specific induction of the transgene is sufficient to generate HD-like phenotypes. Together these results support a protective role of the N17 domain in HD possibly through nuclear export of the pathological 97Q fragment. This new zebrafish model will facilitate pharmacological, genetic, and mechanistic studies of HD.

## Additional files

Additional file 1: Movie S1. HTT-ΔN17-exon1 fish exhibiting normal swimming behavior. (MOV 19875 kb) Additional file 2: Movie S2. A group of HTT-ΔN17-exon1 fish exhibiting normal swimming behavior. (MOV 6872 kb) Additional file 3: Movie S3. A mHTT-ΔN17-exon1 fish exhibiting abnormal movements corresponding to Stage 2. (MOV 11321 kb) Additional file 4: Movie S4. A group of mHTT-ΔN17-exon1 fish exhibiting abnormal movements corresponding to Stage 2 and 3. (MOV 3295 kb) Additional file 5:
**mHTT-**Δ**N17-exon1 L2 develops progressive motor behavioral deficits.** Similar to Line 1, Line 2 exhibits progressive movement problems corresponding to the described three stages of behavioral abnormality, resulting in immobility and death (*n* = 24). (JPEG 238 kb) Additional file 6:
**Ubiquitous HTT-exon1-GFP expression in HTT-exon1 and HTT-**Δ**N17-exon1 lines.** 3D projections of confocal images of brain sections from HTT-exon1 L1 (A-C) and HTT-ΔN17-exon1 L1 (D-F) stained for GFP and HuC. Note the faint nuclear GFP staining in HTT while HTT-ΔN17-exon1 has brighter nuclear GFP. (G) Relative nuclear GFP intensity measurements in HuC^+^ cells comparing HTT-exon1-GFP versus HTT-ΔN17-exon1, *n* = 10 cells each, ***p* < 0.01, students *t*-test. Measurments were made using ImageJ and normalized to average GFP intensity across the entire area. HTT-exon1-GFP was arbitrarily set at 100 %. HTT-ΔN17-exon1 nuclei were approximately 20 % brighter. (JPEG 1556 kb) Additional file 7:
**Quantification of cell and HTT aggregate density in mHTT and mHTT-**Δ**N17 fish brain sections.** (A) Cell density as measured by DAPI positive nuclei per brain volume was not significantly different between the lines. (B) Percent of HuC positive cells over total DAPI positive nuclei is not different between the two lines. (C) GFP^+^ HTT-exon1 aggregate density is not different between the two lines. All comparisons using Student’s *t*-test, *p* < 0.05, not significant (ns). Cells and aggregates were quantified as described in Fig. 4 and Methods. (JPEG 223 kb)
