# Supplementary figures and images for: The N17 domain mitigates nuclear toxicity in a novel zebrafish Huntington’s disease model
Source: Mol Neurodegener. 2015 Dec 9;10:67. doi: 10.1186/s13024-015-0063-2 (PMC4673728; doi:10.1186/s13024-015-0063-2)

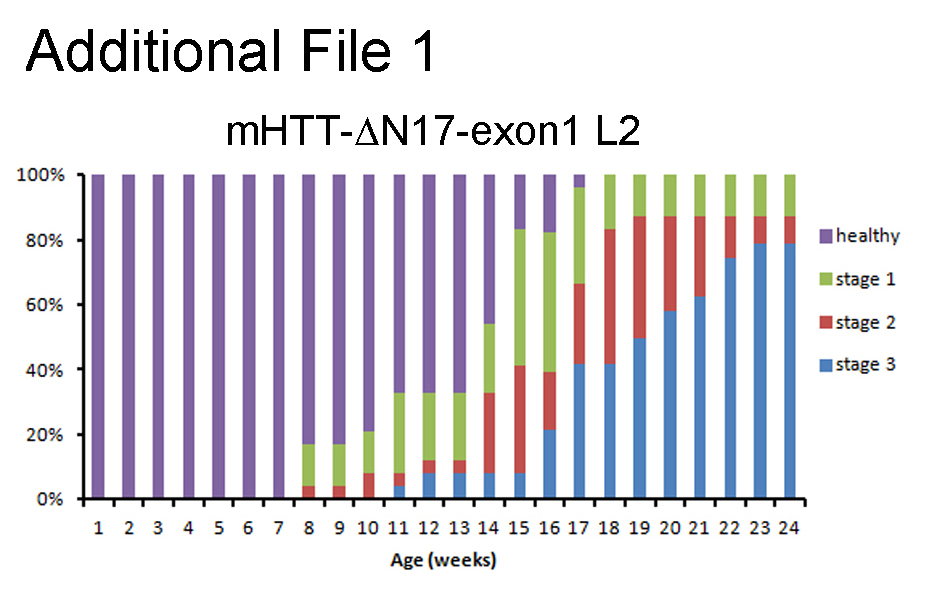

Supplement: Additional file 5: — mHTT-ΔN17-exon1 L2 develops progressive motor behavioral deficits. Similar to Line 1, Line 2 exhibits progressive movement problems corresponding to the described three stages of behavioral abnormality, resulting in immobility and death (n = 24). (JPEG 238 kb) [file 13024_2015_63_MOESM5_ESM.jpeg]

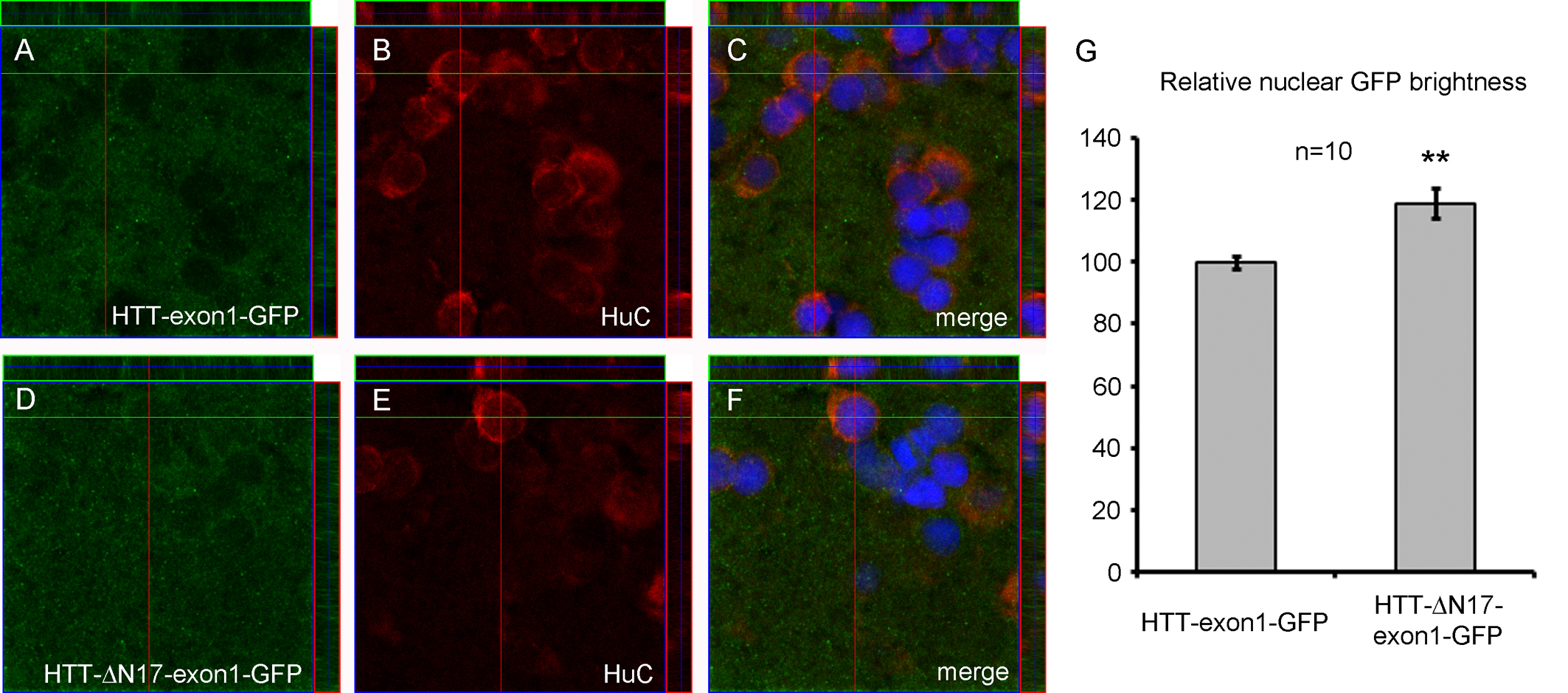

Supplement: Additional file 6: — Ubiquitous HTT-exon1-GFP expression in HTT-exon1 and HTT-ΔN17-exon1 lines. 3D projections of confocal images of brain sections from HTT-exon1 L1 (A-C) and HTT-ΔN17-exon1 L1 (D-F) stained for GFP and HuC. Note the faint nuclear GFP staining in HTT while HTT-ΔN17-exon1 has brighter nuclear GFP. (G) Relative nuclear GFP intensity measurements in HuC+ cells comparing HTT-exon1-GFP versus HTT-ΔN17-exon1, n = 10 cells each, **p < 0.01, students t-test. Measurments were made using ImageJ and normalized to average GFP intensity across the entire area. HTT-exon1-GFP was arbitrarily set at 100 %. HTT-ΔN17-exon1 nuclei were approximately 20 % brighter. (JPEG 1556 kb) [file 13024_2015_63_MOESM6_ESM.jpeg]

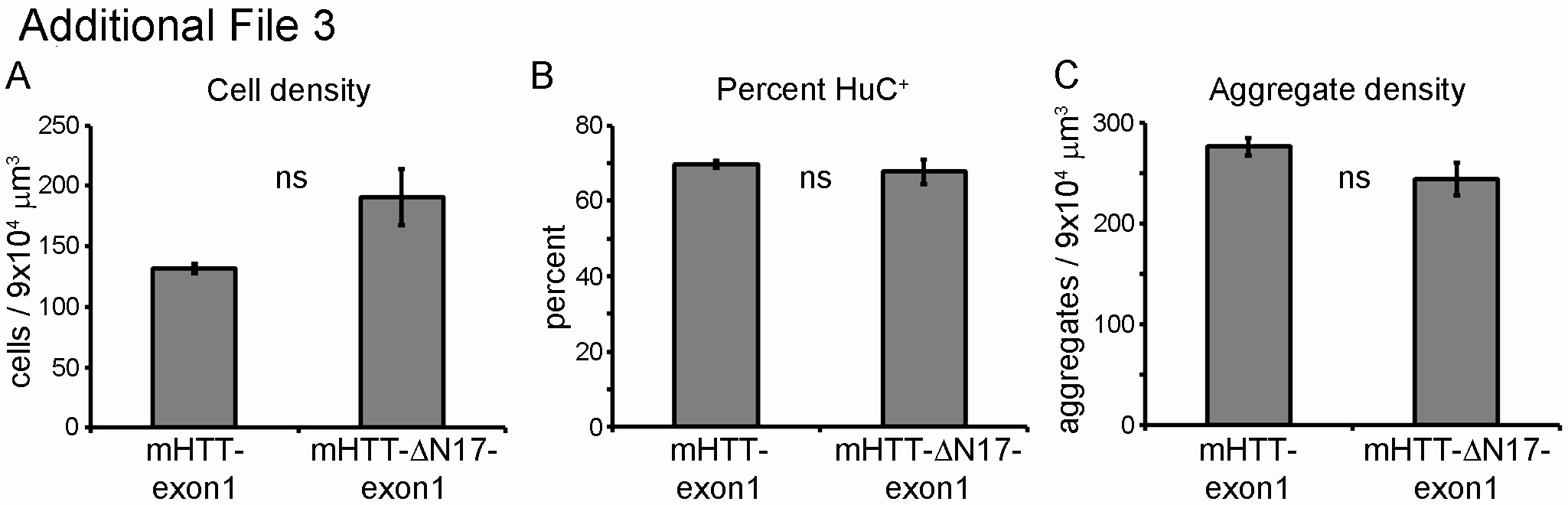

Supplement: Additional file 7: — Quantification of cell and HTT aggregate density in mHTT and mHTT-ΔN17 fish brain sections. (A) Cell density as measured by DAPI positive nuclei per brain volume was not significantly different between the lines. (B) Percent of HuC positive cells over total DAPI positive nuclei is not different between the two lines. (C) GFP+ HTT-exon1 aggregate density is not different between the two lines. All comparisons using Student’s t-test, p < 0.05, not significant (ns). Cells and aggregates were quantified as described in Fig. 4 and Methods. (JPEG 223 kb) [file 13024_2015_63_MOESM7_ESM.jpeg]
